# Targeting Calcitriol Metabolism in Acute Vitamin D Toxicity—A Comprehensive Review and Clinical Insight

**DOI:** 10.3390/ijms251810003

**Published:** 2024-09-17

**Authors:** Simon Aberger, Nikolaus Schreiber, Stefan Pilz, Kathrin Eller, Alexander R. Rosenkranz, Alexander H. Kirsch

**Affiliations:** 1 Division of Nephrology, Department of Internal Medicine, Medical University of Graz, 8036 Graz, Austria; simon.aberger@medunigraz.at (S.A.); nikolaus.schreiber@medunigraz.at (N.S.); kathrin.eller@medunigraz.at (K.E.); alexander.rosenkranz@medunigraz.at (A.R.R.); 2 Department of Internal Medicine I—Nephrology, Paracelsus Medical University, 5020 Salzburg, Austria; 3 Division of Anesthesiology and Intensive Care 2, Department of Anesthesiology and Intensive Care, Medical University of Graz, 8036 Graz, Austria; 4 Division of Endocrinology and Diabetology, Department of Internal Medicine, Medical University of Graz, 8036 Graz, Austria; stefan.pilz@medunigraz.at

**Keywords:** vitamin D, drug toxicity, CYP polymorphism, pharmacogenetics, nutrients, calcitriol

## Abstract

High-dose vitamin D supplementation is common in the general population, but unsupervised high-dose supplementation in vitamin D-replete individuals poses a risk of severe toxicity. Susceptibility to vitamin D toxicity shows a significant inter-individual variability that may in part be explained by genetic predispositions (i.e., CYP24A1 polymorphism). The classic manifestation of vitamin D toxicity is hypercalcemia, which may be refractory to conventional therapy. Its causes include the endogenous overaction of 1α-hydroxylase, monogenic alterations affecting vitamin D metabolizing enzymes and exogenous vitamin D intoxication. In this manuscript, we include a literature review of potential pharmacological interventions targeting calcitriol metabolism to treat vitamin D intoxication and present a case of severe, exogenous vitamin D intoxication responding to systemic corticosteroids after the failure of conventional therapy. Systemic glucocorticoids alleviate acute hypercalcemia by inhibiting enteric calcium absorption and increasing the degradation of vitamin D metabolites but may cause adverse effects. Inhibitors of 1α-hydroxylase (keto/fluconazole) and inducers of CYP3A4 (rifampicin) may be considered steroid-sparing alternatives for the treatment of vitamin D intoxication.

## 1. Introduction

### 1.1. Physiological Background

**Figure 1:**
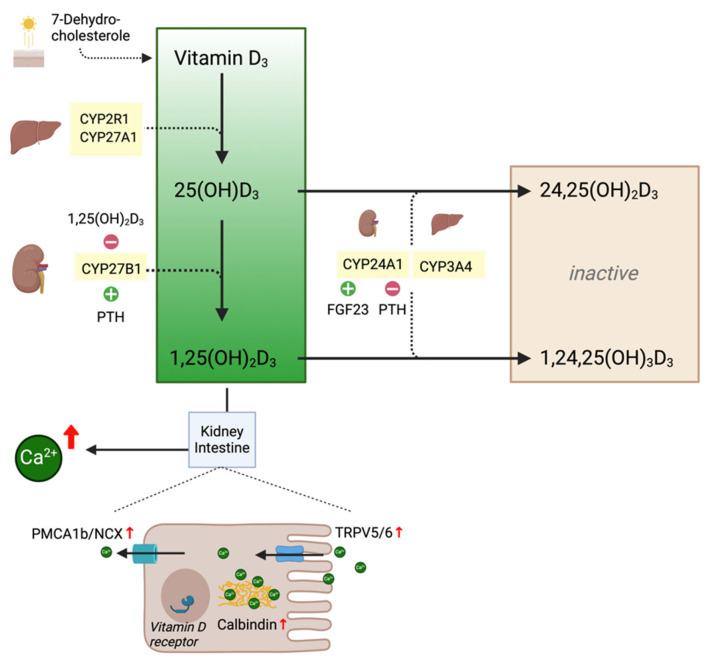
Key enzymes of calcitriol metabolism. Scheme of calcitriol metabolism, including key enzymatic steps of activation and degradation and their respective regulatory mechanisms. Red arrows indicate an increase or upregulation of respective elements.

The synthesis of vitamin D_3_ (cholecalciferol) from cholesterol predecessors and its two-step activation by hepatic 25-hydroxylase to 25-hydroxyvitamin D_3_ (25(OH)D_3_; calcidiol) and renal 1α-hydroxylase to 1,25-dihydroxyvitamin D_3_ (1,25(OH)_2_D_3_; calcitriol) is well characterized (Figure 1). Several enzymes of the cytochrome P450 (CYP) family exert 25-hydroxylase activity. The main hepatic 25-hydroxylase is microsomal CYP2R1. CYP2R1 is weakly controlled by negative feedback from 25(OH)D_3_ but is not known to underlie significant regulatory mechanisms and is therefore not considered a rate-limiting step, even in the presence of supraphysiological 25(OH)D_3_ levels [1]. Additional 25-hydroxylase activity is seen with mitochondrial CYP27A1 [2]. However, murine double-knockout models for neither of the two enzymes resulted in severe rickets, suggesting that 25-hydroxylase activity is pleiotropic, making it an unlikely target for pharmacological interventions [3]. The further conversion of 25(OH)D_3_ to 1,25(OH)_2_D_3_ by 1α-hydroxylase is catalyzed by CYP27B1 in proximal renal tubules and extrarenal sites (i.e., keratinocytes, lymphatic cells). Renal CYP27B1 is stimulated by the parathyroid hormone (PTH), hypocalcemia and hypophosphatemia and receives negative feedback from 1,25(OH)_2_D_3_ as well as hypercalcemia [4]. Extrarenal CYP27B1 activity in lymphoid cells increases in inflammatory states but is counterbalanced by the parallel induction of CYP24A1 to exert only autocrine or paracrine effects in physiologic conditions [5]. Endogenous hypervitaminosis D may develop in sarcoidosis, tuberculosis or fungal infections due to the excessive activation of extrarenal 1α-hydroxylase in macrophages, resulting in systemically elevated 1,25(OH)_2_D_3_ levels [6,7].

1,25(OH)_2_D_3_ increases the expression of the apical calcium channels “transient receptor potential Vanilloid subfamily 6 and 5” (TRPV6 and TRPV5) and the intracellular calbindin and basolateral calcium channels “plasma membrane calcium ATPase” (PMCA1b) and “Sodium-Calcium exchanger” (NCX) to facilitate the transcellular absorption of ionized calcium molecules in intestinal and renal proximal tubular cells [8]. The net effect on bone mineralization is dependent on the combined effect of calcium and PTH plasma levels, but, in the setting of high 1,25(OH)_2_D_3_, the increased receptor activation of NFκB (RANK) expression favors osteoclast differentiation to mobilize calcium from bone [8]. 1,25(OH)_2_D_3_ is highly biologically active and exerts its long-lived genomic effects via nuclear receptors, while 25(OH)D_3_ is thought to have negligible biologic effects. However, more recent studies suggest that 25(OH)D_3_ can overpower 1,25(OH)_2_D_3_ through the competitive binding of nuclear vitamin D receptors at high enough concentrations and may exert both systemic and paracrine effects [9,10]. This notion was supported by a murine CYP27B1^−/−^ model, showing equal dose-dependent toxicity when exposed to 25(OH)D_3_ and 1,25(OH)_2_D_3_ [11]. As sterol derivates, 25(OH)D_3_ and 1,25(OH)_2_D_3_ are highly hydrophobic/lipophilic and are, therefore, transported by vitamin D binding protein (VDBP) in blood plasma, with the potential to accumulate in adipose tissue, prolonging their half-lives for up to a few months [9,12].

### 1.2. Clinical Background

The biologic effect of supplemental vitamin D on general health, infectious diseases and chronic inflammatory diseases has been at the forefront of research over the past two decades [13]. Recently, the interest in vitamin D supplementation has been spiking in the general population due to the SARS-CoV-2 pandemic and claims that supplemental vitamin D may improve COVID-19 outcomes [14]. Short-term high-dose vitamin D supplementation is gaining popularity for treating acute and chronic inflammatory diseases such as COVID-19 [15] and demyelinating neurologic diseases [16], respectively. However, the potential benefits and risks of supplementation in vitamin D-replete individuals have been the subject of ongoing debate [13,17]. Nutritional supplements have increasingly been promoted on social media without appropriate peer review, education and regulation. The target range of 25(OH)D_3_ is commonly referenced as being 30–80 ng/mL [18], and supplementation is recommended in cases of deficiency [19]. However, the unsupervised intake of supplements may harbor a risk for serious complications, especially in vitamin D-replete individuals. General recommendations suggest a maximum daily dose of supplemental vitamin D (typically vitamin D_3_, cholecalciferol) of 4000 international units (IU; [40 IU = 1 µg]) for adults, harboring only a minimal risk of adverse effects [18], but no exact toxicity threshold or toxic cumulative dose is known [20]. The potential risk of sustained supplementation above this recommended daily dose is not well defined, but three percent of the general population in the US exceeded the tolerable upper limit of 4000 IU/day [21]. While single doses >100,000 IU of vitamin D_3_ are tolerated without adverse events [22], the current literature describes sustained high-dose vitamin D_3_ supplementation regimens >100,000 IU daily for several months as having an 11% to 20% incidence of hypercalcemia, but susceptibility to adverse events seems to vary individually [23,24]. Even vitamin D_3_ doses of 3200–4000 IU per day in trials lasting at least six months appear to increase the risk of hypercalcemia and some other adverse events in a small proportion of individuals [18]. Responsiveness to vitamin D_3_ supplementation in vitamin D-deficient individuals is related to polymorphisms in the vitamin D receptor (VDR), *CYP2R1* and *CYP24A1* genes [25]. Therefore, the heterogenous interindividual susceptibility to vitamin D toxicity may similarly depend on genetic predispositions in CYP metabolism [26], and secondary factors such as immobilization, acute illness, comorbidities and comedications, may trigger hypercalcemic episodes in predisposed individuals [27].

Vitamin D toxicity is clinically characterized by prolonged, severe and parathyroid hormone (PTH)-independent hypercalcemia resulting from the increased intestinal absorption of calcium and mobilization of calcium from bone. Its etiologies include an excessive intake of vitamin D and increased 1α-hydroxylase activity in granulomatous disease [9,28] or genetic diseases such as the loss of function of catabolic 24-hydroxylase (CYP24A1) [29] resulting in endogenous hypervitaminosis D [30]. Acute manifestations of vitamin D toxicity include acute kidney injury, cardiac arrhythmia and neuropsychiatric disease, but chronic sequelae such as kidney stones, gastric ulcers and chronic kidney disease with nephrocalcinosis may also develop, as well as potential adverse effects unrelated to calcium metabolism [31]. The available guidelines for the treatment of hypercalcemia mainly refer to malignancy-associated hypercalcemia and include regimens for the use of intravenous fluids, bisphosphonates, denosumab, calcitonin and glucocorticoids [32]. However, there are currently no evidence-based guidelines for the treatment and follow-up of vitamin D-mediated hypercalcemia due to high-dose supplementation, hampering structured clinical decision making [33].

In general, evidence of exact toxicity thresholds, adequate therapy and long-term follow-up in patients with acute vitamin D toxicity are limited. Herein, we present a comprehensive literature review concerning potential pharmacological interventions into calcitriol metabolism and the clinical management of vitamin D toxicity alongside a case presentation of hypercalcemia complicated by acute kidney injury due to excessive and prolonged vitamin D supplementation.

## 2. Methods

Our literature review of pharmacological interventions into calcitriol metabolism to treat vitamin D toxicity is based on a PubMed search for articles published between 1980 and 2024. The search was conducted on 5 May 2024 using specific MESH terms, including the following: “vitamin D toxicity CYP3A4” OR “vitamin D toxicity CYP24A1” OR “vitamin D toxicity CYP27B1” OR “hypercalcemia CYP”. After screening the titles and abstracts of the 128 retrieved publications, 73 studies were excluded due to non-relation to the subject, 13 studies were excluded due to non-retrievable manuscripts, and a total of 42 studies were finally included for detailed review and discussion. To provide additional clinical insight, we discuss a detailed case report of acute hypercalcemia caused by exogenous vitamin D intoxication, which responded to systemic glucocorticoid treatment. Figure preparation was conducted with Prim10 (GraphPad, San Diego, CA, USA) and BioRender (Biorender.com ©2024).

## 3. Review—Targeting Calcitriol Metabolism

In cases of vitamin D intoxication, the cessation of vitamin D supplementation and the restriction of calcium intake are rational measures, but insufficient to control the acute side effects of hypercalcemia. Due to the long half-lives of vitamin D metabolites, hypercalcemia can be refractory to fluids, diuretics and antiresorptive therapies such as bisphosphonates or denosumab. To facilitate the elimination of vitamin D metabolites in cases of intoxication, it may be practical to inhibit vitamin D synthesis and promote its catabolism.

### 3.1. Degradation of Vitamin D Metabolites

**Figure 2:**
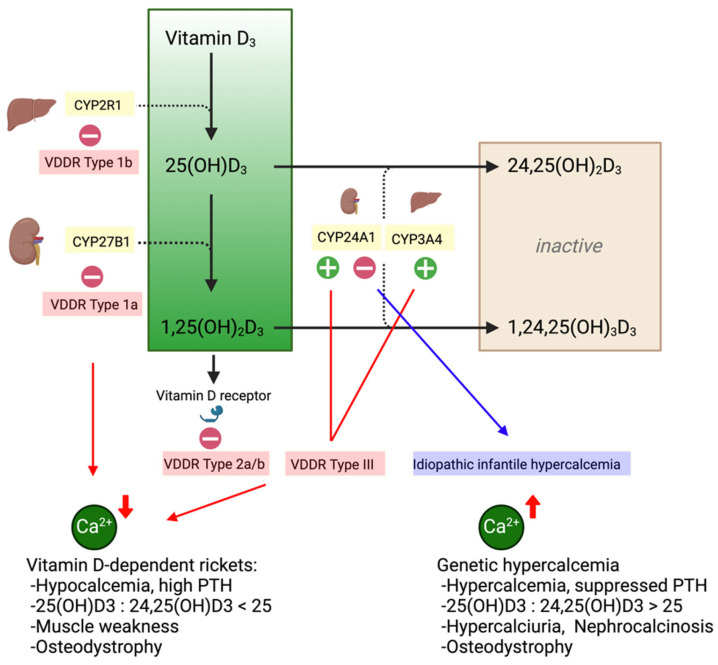
Genetic conditions affecting calcitriol metabolism. Gain/loss-of-function mutations in the genes of key enzymatic steps of calcitriol metabolism can cause vitamin D-dependent rickets (VDDR), and defective calcitriol degradation by CYP24A1’s loss of function can cause idiopathic infantile hypercalcemia (IHH).

The elimination of systemic 25(OH)D_3_ and 1,25(OH)_2_D_3_ is less well studied but involves 24-hydroxylase (CYP24A1) in renal tubular cells degrading both substrates to biologically inactive and water-soluble 1,24,25(OH)_3_D_3_ and 24,25(OH)_2_D_3,_ respectively [34]. The intrinsic activity of renal CYP24A1 is low, but both 1,25(OH)_2_D_3_ and fibroblast growth factor 23 (FGF-23) stimulate CYP24A1 activity, while it is suppressed in the presence of PTH [34,35]. The additional degradation of 25(OH)D_3_ and 1,25(OH)_2_D_3_ is catalyzed by hepatic CYP3A4, which is not directly regulated by vitamin D metabolites or PTH but may be inducible by certain drugs [36]. A genetic predisposition to the dysregulation of calcitriol metabolism has been exemplified by the biallelic mutational loss of the 24-hydroxylase activity of CYP24A1, resulting in idiopathic infantile hypercalcemia (Figure 2). Idiopathic infantile hypercalcemia (IHH) is characterized by the uncontrolled production of 1,25(OH)_2_D_3_ with severe, complicated hypercalcemia, hypercalciuria and nephrocalcinosis [30,37,38]. More recently, monoallelic mutations and polymorphisms affecting CYP24A1 activity have been implicated in a subset of patients diagnosed with idiopathic hypercalciuria who are predisposed to nephrolithiasis and nephrocalcinosis, but the clinical picture is less severe than with IHH [39]. Among these patients, CYP24A1 activity translated into a shift of the vitamin D metabolite diagnostic ratio of 24,25(OH)_2_D_3_ to 25(OH)D_3_, with 25(OH)D_3_: 24,25(OH)_2_D_3_ > 25 [40]. Large-scale population-based research programs are currently planned and will investigate whether polymorphisms in the genes of key enzymes, such as CYP2R1, CYP27B1, CYP24A1 and VDBP, may explain the overall heterogenous connections between vitamin D metabolism, the response to supplementation and its effects on bone health [41].

### 3.2. Therapeutic Targets

**Figure 3:**
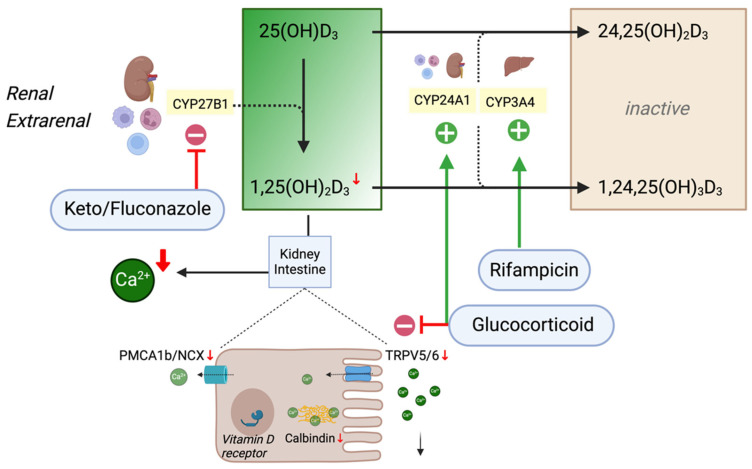
Pharmacological targets of calcitriol metabolism in renal and extrarenal tissue. The inhibition and induction of key enzymatic steps in the activation and degradation of vitamin D metabolites, leading to a decrease in 1,25(OH)_2_D_3_. Red arrows indicate a decrease or downregulation of respective elements.

Based on this molecular background, potential therapeutic targets for decreasing biologically active calcitriol levels may include CYP27B1 inhibition and the induction of renal and extrarenal CYP24A1, as well as hepatic CYP3A4. Additionally, specific VDR antagonism may be a potent option to reverse the acute effects of calcitriol. In the following, we present the current evidence on how pharmacological agents influence the activity of these enzymes, offering potential treatment options for vitamin D intoxication (Figure 3).

#### 3.2.1. Glucocorticoids

Glucocorticoids have been shown to downregulate the active intestinal absorption of calcium by reducing the expression of TRPV6, calbindin and PMCA1b in experimental studies, directly counteracting the effects of 1,25(OH)_2_D_3_ [42]. Dexamethasone, in the presence of 1,25(OH)_2_D_3_, was shown to increase 24-hydroxylase mRNA expression in isolated osteoblasts and renal tubular cells, enhancing 1,25(OH)_2_D_3_ degradation in vitro [43]. The application of dexamethasone in vitamin D-replete mice causes upregulation of renal CYP24A1 [44], facilitated by crosstalk between the glucocorticoid receptor and the vitamin D-receptor-mediated promotion of CYP24A1 expression [45]. This shift from 1α-hydroxylation to 24-hydroxylation was also observed in dexamethasone-treated leukocytes [46], suggesting additional effects of glucocorticoids on extrarenal calcitriol metabolism. A negative impact on bone density is a well-known side effect of long-term systemic glucocorticoid therapy, warranting the prophylactic supplementation of vitamin D and calcium in this setting [47].

In the context of vitamin D-mediated hypercalcemia, glucocorticoids may be used to decrease intestinal calcium absorption to correct serum calcium levels, while the induction of CYP24A1 may help to decrease active vitamin D metabolites’ half-lives (Figure 3). However, systemic glucocorticoids may be required for longer periods of time to achieve the latter, which increases the risk of side effects. Enteric budesonide is well tolerated, has an improved safety profile and has been shown to decrease enteric cell calcium absorption in an experimental study [48]. Despite its low resorptive capacity, budesonide may still exert some systemic effects, especially on the liver, since its rudimentary resorption is likely to undergo hepatic first-pass metabolism [49]. While the use of an enteric steroid may be an intriguing option to reduce systemic glucocorticoid exposure, its use has, to our knowledge, not yet been reported in clinical scenarios involving vitamin D toxicity.

#### 3.2.2. CYP27B1 Inhibition

The biallelic mutation of CYP27B1 is clinically correlated to vitamin D-dependent hypophosphatemic rickets type I [50] (Figure 2), suggesting insufficient alternative pathways to compensate for the loss of this enzymatic step [51]. The antimycotic drug ketoconazole is known to inhibit sterol synthesis and has been shown to inhibit the 1α-hydroxylase activity of CYP27B1 in isolated human renal tubular cell lines in vitro [52]. Ketoconazole has been applied in clinical scenarios of steroid refractory hypercalcemia in sarcoidosis due to its allegedly higher potency for extrarenal CYP27B1 inhibition [53]. This effect has been demonstrated ex vivo by exposing isolated pulmonary macrophages from a patient with sarcoidosis to ketoconazole, leading to a marked decrease in enzymatic 1,25(OH)_2_D_3_ production [54]. Hydroxychloroquine is thought to exert similar effects on mononuclear cells, but its successful use in sarcoidosis-related hypercalcemia has been reported only in a single case [55]. Furthermore, the treatment of CYP24A1-deficient patients with ketoconazole [56] and fluconazole [57,58] alleviated hypervitaminosis D, leading to the normalization of their serum calcium and hypercalciuria.

#### 3.2.3. CYP3A4 Induction

Gain-of-function mutations affecting CYP3A4 activity have previously been identified by whole-genome sequencing in patients with vitamin D-dependent rickets type 3 (Figure 2), showing an increased conversion of 25(OH)D_3_ and 1,25(OH)_2_D_3_ into their inactive forms [59], which exemplifies the enzyme’s potency in degrading vitamin D. The induction of hepatic CYP3A4 can be potently achieved by rifampicin [60]. Ex vivo data suggest up to an 80-fold increase in CYP3A4 mRNA expression in human hepatocytes after stimulation with rifampicin [61]. CYP3A4 then inactivates 25(OH)D_3_ and 1,25(OH)_2_D_3_, similar to 24-hydroxylation, as previously discussed. The successful use of rifampicin to provide an alternative pathway of vitamin D catabolism has been reported in patients with an impaired function of their CYP24A1, which is linked to idiopathic hypercalcemia, significantly improving serum calcium and hypercalciuria [62]. Since rifampicin potently influences hepatic enzyme activity, it may be of interest in increasing the elimination of vitamin D metabolites in exogenous vitamin D intoxication. However, its use has yet to be proven in this scenario.

#### 3.2.4. VDR Antagonists

Certain modulators of the VDRs (vitamin D analogues) can exert actions as a VDR antagonist and are therefore considered a promising therapeutic option for the treatment of vitamin D intoxication [63,64]. In this context, the VDR antagonist ZK168281 was shown to successfully treat calcitriol-induced hypercalcemia in mice, but clinical data in humans are still missing [65].

In this regard, we would like to present a case of exogenous vitamin D intoxication complicated by hypercalcemia and discuss our therapeutic approach and clinical follow-up data in the context of our literature review.

### 3.3. Case Presentation

A 52-year-old Caucasian man with a history of neuromyelitis optica presented to the emergency department with worsening headache, nausea and anorexia. His premedication included mycophenolate, calcium supplements and an over-the-counter vitamin D supplement containing cholecalciferol. The patient’s vital signs and physical examination were unremarkable, and his ECG did not show any arrhythmia or QT-interval shortening. Initial laboratory tests revealed acute kidney injury (AKI stage II) with elevated serum creatinine levels of 1.5 mg/dL (132 µmol/L) and severe hypercalcemia (total serum calcium level was 3.66 mmol/L, ionized calcium level was 1.82 mmol/L, with a normal serum albumin level of 4.0 g/L and a normal serum phosphate level of 1.0 mmol/L). The initial treatment included volume expansion with 3 L of intravenous crystalloids and 40 mg of furosemide to maintain the urine output at 100–150 mL/h, and all oral supplements were discontinued along with an additional restriction of dietary calcium. Despite adequate rehydration and urine output, the patient’s calcium level increased to 3.9 mmol/L (ionized calcium level 1.98 mmol/L) after approximately 12 h. A single dose of zoledronate was administered, and therapy with calcitonin was initiated at a dose of 8 Units/kg every 8 h.

**Figure 4:**
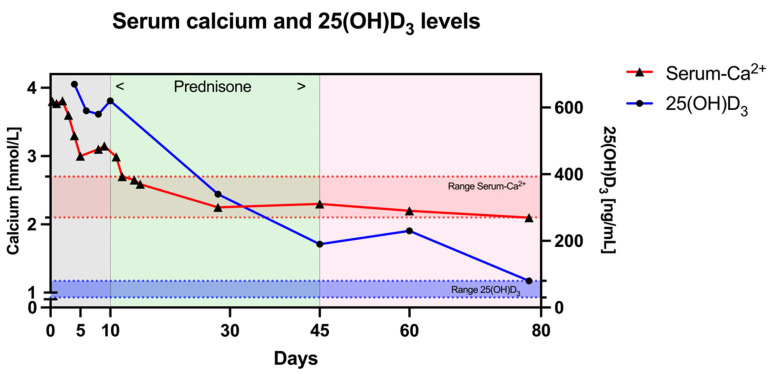
Case report: total serum calcium and 25(OH)D_3_ levels over time. Total serum calcium levels (red) in mmol/L (normal range 2.2–2.65 mmol/L, red dotted lines) and 25-hydroxy-vitamin-D-levels (blue) in ng/mL (normal range 30–80 ng/mL, blue dotted lines) are shown over the time in days. Timeframes of treatment with normal saline, furosemide, calcitonin and zoledronate (gray), subsequent use of prednisone (green) and follow-up (pink) are shown. Conversion of 25(OH)D_3_ is 1 ng/mL = 2.5 nmol/L.

The patient, however, displayed an inadequate response to this therapy, and his calcium levels remained elevated after six days of hospitalization, about 72 h after bisphosphonate application and starting with calcitonin (Figure 4), with his peak creatinine reaching 2.57 mg/dL (226.2 µmol/L). A renal ultrasound did not reveal any structural abnormalities, and no proteinuria was present alongside unremarkable urinary sediment. His intact PTH level was suppressed, at <5 pg/mL, with severely elevated 25(OH)-D_3_ levels, at 548 ng/mL. A detailed medical history revealed that the patient had been taking roughly 5 mL of cholecalciferol 36,000 IE/mL per day for the last five months due to a lack of information about proper dosing, combined with oral calcium supplementation above 1000 mg daily, in addition to a regular Western diet.

Due to a lack of response, the patient was started on 1 mg/kg of prednisone. Less than 48 h later, his total serum calcium and ionized calcium levels finally started to decrease and reached the normal range after 12 days of hospitalization, when the patient was discharged. A PET-CT scan showed no signs of neoplastic or granulomatous inflammatory activity. Serum and urine electrophoresis with immunofixation displayed no evidence of monoclonal gammopathy. Creatinine levels had decreased to 1.55 mg/dL at day 12, while 25(OH)D_3_ levels were still elevated (around 500 ng/mL) at the time of discharge.

During consecutive outpatient follow-up appointments, his calcium levels further decreased on day 15, allowing for the weekly tapering of prednisone by −5 mg. His serum calcium finally stabilized within the normal range on day 27. Additionally, the patient’s creatinine level returned to baseline, and his 25(OH)D_3_ levels were first noted to significantly decrease between day 15 and day 27. Prednisone was stopped after 6 weeks. Calcium levels remained stable thereafter, while 25(OH)D_3_ levels rebounded once to 220 ng/mL on day 60 (14 days after steroid cessation) before decreasing to <150 ng/mL without additional interventions. An ultrasound did not show any evidence of nephrocalcinosis; a 24 h urine calcium excretion, measurement of 24,25(OH)_2_D_3_ and CYP genotyping were not performed in this case.

## 4. Discussion

Vitamin D toxicity appears to be a rare event, even though vitamin D supplements are being increasingly used by the general population [21,66]. As reviewed by Galior et al., cases of hypercalcemia due to the over-supplementation of exogenous vitamin D_3_ have typically included daily doses above 50,000 IU over at least 2–3 months, with treatment approaches including intravenous fluids, loop diuretics, calcitonin, bisphosphonates and glucocorticoids, with the latter used mostly in pediatric cases [67,68]. The exact relationship between vitamin D_3_’s dosage, plasma level and biologic effect is still elusive; however, adverse effects with hypercalciuria followed by hypercalcemia seem to increase with 25(OH)D_3_ levels beyond 150 ng/mL [31]. In titration studies, prolonged ultra-high-dose vitamin D_3_ supplementation (>100,000 IU daily) with sustained 25(OH)D_3_ plasma levels above 300 ng/mL showed a 20% incidence of hypercalcemia [23], whereas short-term plasma peaks around 400 ng/mL with daily doses between 10,000 and 40,000 IU daily were tolerated without complications [69]. This suggests that vitamin D toxicity mainly correlates with sustained high plasma levels, whereas shorter plasma peaks may be better tolerated. In our patient, we calculated a daily dose of 150,000 IU over at least 3 months, reaching sustained plasma levels beyond 500 ng/mL, which compares best to ultra-high-dose titration data and exemplifies the missing negative feedback regulation of 25-hydroxylase activity. Another possible explanation of interindividual variability in response to vitamin D supplementation involves genetic polymorphisms affecting vitamin D degradation, mainly CYP24A1 24-hydroxylase activity. Vitamin D-mediated hypercalcemia is a known trait of CYP24A1 loss-of-function mutations [40]; however, its presence and genetic variability have still to be determined in large-scale population-based studies. Among cases of vitamin D-sensitive hypercalcemia in children, a 25(OH)D_3_:24,25(OH)_2_D_3_ ratio > 25 with upregulated alternative metabolism via 3-epi-25(OH)D_3_ has been described to clinically identify CYP24A1’s loss of function [68]. Similarly, polymorphisms affecting CYP24A1 have been implicated in the idiopathic hypercalciuria phenotype, which is predisposed to nephrolithiasis and nephrocalcinosis, with increased susceptibility to vitamin D-mediated hypercalcemia [39]. Therefore, screening for CYP24A1 deficiency might be warranted before high-dose vitamin D supplementation, potentially by using the 25(OH)D_3_:24,25(OH)_2_D_3_ diagnostic ratio.

As discussed previously, at supraphysiological levels, 25(OH)D_3_ likely surpasses the binding capacity of vitamin D binding protein (VDBP), leading to an increase in free 25(OH)D_3_ and other vitamin D metabolites in circulation, stimulating enteral calcium absorption. The coadministration of oral calcium supplements likely further amplified increased enteral calcium absorption in our case. Since hypercalcemia was refractory to standard therapy, systemic glucocorticoids had to be considered, as they have been shown to reduce intestinal calcium absorption in the current literature [70]. It should be noted that these recommendations are based on animal studies and oncologic cohort studies using glucocorticoids to treat malignancy-associated hypercalcemia [71,72]. After an appropriate risk–benefit evaluation, we administered 1 mg/kg prednisone to our patient, and his serum calcium levels normalized within 4 days (Figure 4).

As a fat-soluble vitamin, vitamin D_3_ has a high distribution volume due to its accumulation in liver, muscle and fat tissue without any alternative elimination pathway other than enzymatic metabolization [9]. This was most likely reflected by the slow decrease in 25(OH)D_3_ plasma levels over several weeks despite the rapid normalization of serum calcium within days of the administration of oral glucocorticoids (Figure 4). Therefore, glucocorticoids were tapered slowly over 6 weeks until 25(OH)D_3_ plasma levels decreased below 150 ng/mL. Interestingly, 25(OH)D_3_ plasma levels started to briefly rise again 14 days after glucocorticoid cessation, suggesting a decrease in 25(OH)D_3_ degradation after the fading of the steroid effect, potentially aggravated by its release from adipose tissue or other tissues with weight loss. Since serum calcium did not increase again, no additional use of glucocorticoids was deemed necessary. However, such relapses have been reported in other cases, prompting the use of additional strategies to control serum calcium levels [53]. In an experimental study, enteral steroids were shown to decrease intestinal calcium absorption [48], and the use of ketoconazole, fluconazole and rifampicin has recently been reported in cases of endogenous vitamin D toxicity due to CYP24A1’s gain of function [56,57,62]. Therefore, the use of enteral budesonide, keto/fluconazole or rifampicin may be a steroid-sparing strategy worth investigating in patients with relapsing or refractory hypercalcemia due to vitamin D toxicity after a shortened course of high-dose glucocorticoids to alleviate acute hypercalcemia.

From a public health perspective, vitamin D supplementation is increasing in popularity among the general population since unfiltered health advice can be accessed on the internet. Notably, our patient suffered from neuromyelitis optica, which is a demyelinating neurologic disease adjacent to the multiple sclerosis spectrum. This closely connects our case to the controversial topic of high-dose vitamin D supplementation among patients with multiple sclerosis [73]. The role of vitamin D deficiency in the pathophysiology of multiple sclerosis and its supplementary correction as a therapeutic strategy have been expertly reviewed by Feige et al., who concluded that there is still no solid evidence of a benefit from vitamin D supplementation exceeding general dosing recommendations [73]. Therefore, a detailed understanding of the vulnerability in this patient group is needed for physicians to provide appropriate patient information and competent therapy guidance [74].

Significant knowledge gaps remain concerning the management of non-malignant hypercalcemia, since most guidelines derive from oncologic studies and mechanistic rationales. The efficacy of enteral budesonide in decreasing intestinal calcium absorption and CYP3A4’s induction by rifampicin may be worth investigating as steroid-sparing alternatives, whereas the use of keto/fluconazole to inhibit CYP27B1 may be more potent in cases with endogenous calcitriol toxicity, following a pathophysiological rationale. Therefore, further research should be dedicated to experimental studies and comprehensive clinical research to improve our understanding and management of vitamin D-associated hypercalcemia.

Finally, the popularity of supplementation with over-the-counter medications is rising and, therefore, pharmacists as well as physicians and the public media are responsible for informing the public about correct dosing and potential health hazards, especially in vulnerable patient groups using high-dose supplementation [74]. The genetic background of the striking interindividual variance in responses to vitamin D supplementation is intriguing. Recessive CYP24A1 mutations and polymorphisms facilitating the slow metabolism of calcitriol have been gaining attention in recent observational studies and may in part explain the heterogenous susceptibility to vitamin D toxicity. Pharmacogenetic testing of CYP metabolism may be conducted in individuals with suspected vitamin D-mediated hypercalcemia alongside careful differential diagnostics, including the 25(OH)D_3_:24,25(OH)_2_D_3_ ratio.

In conclusion, vitamin D toxicity is an important differential diagnosis in patients with PTH-independent hypercalcemia and can be refractory to standard therapy. A diagnostic classification is still ill defined but should include the measurement of elevated 25(OH)D_3_ and a conclusive patient history to ascertain high-dose supplementation far beyond 4000 IU daily (usually >50,000 IU of vitamin D_3_ daily) for several months, with the exclusion of alternative causes. In our patient, hypercalcemia was refractory to standard therapy and rapidly responded to 1 mg/kg prednisolone, tapering over 6 weeks until their serum 25(OH)D_3_ level was below 150 ng/mL. Due to the longevity of vitamin D metabolites in vivo, the monitoring of 25(OH)D_3_ levels as well as serum calcium levels should be conducted for at least 6 months to identify potential toxicity relapses.

## Data Availability

The authors confirm that the data supporting the findings of this study are available within the article.
